# Clinical characteristics of Guillain–Barré syndrome in patients with primary Sjögren’s syndrome

**DOI:** 10.1038/s41598-024-56365-y

**Published:** 2024-03-09

**Authors:** Xiaoyu Cao, Juan Guo, Yaran Yang, Zhibo Yu, Hua Pan, Wei Zhou

**Affiliations:** 1 Department of Rheumatology and Immunology, Beijing Tiantan Hospital, Capital Medical University, Beijing, China; 2 Department of Neurology, Beijing Tiantan Hospital, Capital Medical University, Beijing, China

**Keywords:** Diseases, Medical research, Neurology

## Abstract

To investigate the clinical characteristics of Guillain–Barré syndrome (GBS) in patients with primary Sjögren’s syndrome (SS). Records of patients with positive anti-SSA antibodies hospitalized in the Beijing Tiantan Hospital between December 2011 and May 2020 were retrieved. Patients who fulfilled the criteria for diagnosis of GBS and primary SS were included, and their clinical data were analyzed. Among the 785 patients with positive anti-SSA, 52 patients were identified in this study. They were 27 males and 25 females with median age of 59 years old. Besides anti-SSA antibodies, multiple autoantibodies were detected in these patients including antinuclear antibody, anti-Ro52, anti-mitochondrial M2, anti-thyroid peroxidase and anti-thyroglobulin autoantibodies. Preceding infection was reported in 42 patients. Hyporeflexia/areflexia and limbs weakness were the most common manifestation and 35 patients presented cranial nerve injuries. GBS disability score of 3, 4 and 5 was scaled in 28 (53.8%), 15 (28.8%) and 3 (5.8%) patients respectively. Forty-six patients received intravenous immunoglobulin (IVIG) monotherapy, 5 patients were treated by IVIG plus glucocorticoids, and 51 patients improved during hospitalization. The frequency of male gender among the patients with both GBS and primary SS suggests an independent onset of GBS and the co-existence of these autoimmune diseases in patients with multiple autoantibodies. Majority of patients with GBS and primary SS experience benign disease course.

## Introduction

Primary Sjögren’s syndrome (SS) is a systemic autoimmune disease characterized by lymphocyte infiltration into the exocrine glands. Xerostomia and xerophthalmia are common manifestations of primary SS. The extra-glandular manifestations of primary SS are variable^1^. SS associated with other autoimmune diseases such as systemic lupus erythematosus, systemic sclerosis, and rheumatoid arthritis is considered as secondary SS^2^. As different systemic autoimmune diseases may involve nerve system by different mechanisms, studies focusing on primary SS should reveal neuropathies caused by abnormal autoimmune reactions of SS. Notably, the primary SS-related neurological clinical spectrum is extensive. The central nervous system, peripheral nervous system (PNS), and autonomous nervous system may be involved. The manifestations of PNS involvement in primary SS include distal axonal sensory polyneuropathy, small-fiber neuropathy, sensorimotor polyneuropathy, multiple mononeuropathy, sensory ganglionopathy, cranial nerve neuropathies, chronic inflammatory demyelinating polyneuropathy (CIDP)^3^.

Nerve conduction studies have shown that primary SS-related peripheral neuropathies can manifest as axonal sensorimotor polyneuropathy and demyelinating polyradiculoneuropathy^4^. Carvajal et al. studied 392 patients with primary SS to determine the prevalence of neurological presentations. They reported that about 15% of patients with primary SS present with PNS involvement^5^. Furthermore, in a study including 1,695 primary SS patients, the prevalence of PNS was 3.7%^6^. A recent publication reported, up to 38% primary SS patient had PNS involvement^7^. The literature varies regarding nervous system involvement of primary SS. This is partly because neurological involvements are often not assessed by neurology specialists. However, Guillain–Barré syndrome (GBS) was not reported in these studies^5–7^. One study identified 44 patients with primary SS from 184 patients who had polyneuropathy with limb weakness, 3 patients were diagnosed as GBS but subtype of GBS was not mentioned in the research^8^. A total of 11 patients suffered from both primary SS and GBS were found in literature as case reports (Table 1)^9–17^. The anti-SSA antibody was positive in seven of the eleven patients. The female-to-male ratio was 9:2.

**Table 1:** The characteristics of primary SS and GBS based on the previously published case reports.

Case	Age	Sex	Subtypes of GBS	Clinical features of SS	Treatment	Outcome
1^9^	40	Female	NA	Positive ANA and anti-SSA/SSB, ultrasound of the parotid glands revealed nonhomogenous glandular tissue	IVIG and GC	Recovery
2^10^	63	Female	AMSAN	Positive ANA and anti-SSA/SSB, keratoconjunctivitis sicca, corneal ulcers with severe superficial punctate keratitis,	IVIG and GC	Recovery
3^11^	41	Female	Demyelinating sensorimotor polyradiculoneuropathy	Fatigue, polyarthralgia, recurrent oral ulcers, difficulty in swallowing food, anaemia, severe leucopenia, positive ANA, early loss of dentures, no uptake in parotid scintigraphy, positive Schirmer’s test ,and lymphocytic infiltration of minor salivary glands	TPE and GC	Recovery
4^12^	82	Female	AIDP	Positive anti-SSA and anti-SSB antibodies, dry eyes and mouth. Abnormal findings from parotid scintigraphy, minor salivary gland biopsy, and Schirmer’s test were compatible with SS	TPE, IVIG, and HCQ	Recovery
5^13^	26	Male	AMAN	Positive ANA and anti-SSA antibodies, biopsy of small salivary glands was consistent with SS	TPE and IVIG	Recovery
6^14^	57	Male	Acute motor dominant demyelinating and axonal neuropathy, and could not classify neuropathy into either AMAN or AIDP	Xerostomia, xerophthalmia, positive anti-SSA antibody, positive results of Schirmer’s test and a salivary gland biopsy	IVIG and GC	Improved
7^15^	67	Female	Acute pure motor neuropathy, which is similar to axonal type GBS	Xerostomia, xerophthalmia, positive ANA and anti-SSA antibody, positive results of Schirmer’s test and a salivary gland biopsy, salivary scintigraphy revealed strong dysfunction. A sialogram of the parotid gland revealed punctate collections of contrast medium (more than 1 mm)	IVIG and GC	Improved
8^16^	42	Female	AMSAN	Xerostomia, xerophthalmia, positive anti-SSA antibody, abnormal salivary scintigraphy, positive results of Schirmer’s test and a salivary gland biopsy	IVIG, GC, and HCQ	Improved
9^17^	40	Female	Acute GBS*	NA	TPE, AZA after pulse CYC	Recovery
10^17^	39	Female	Acute GBS*	NA	TPE, AZA and GC after pulse CYC	Recovery
11^17^	64	Female	Acute GBS*	NA	IVIG, GC, and HCQ	Recovery

*Of 3 patients with acute GBS, 2 patients had acute demyelinating type, and 1 was with motor axonal-type polyradiculoneuritis.

*TPE* therapeutic plasma exchange, *IVIG* intravenous immunoglobulin, *AZA* azathioprine, *CYC* cyclophosphamide, *GC* glucocorticoid, *HCQ* hydroxychloroquine.

Symmetric extremity weakness is a typical clinical feature of GBS. In addition, GBS may present with paresthesia, numbness, pain, involvement of the cranial nerve, and autonomic dysfunction. The major subtypes of GBS are acute inflammatory demyelinating polyneuropathy (AIDP) and acute motor axonal neuropathy (AMAN)^18,19^. Epidemiological studies have revealed the annual incidence of GBS to be 0.8–1.9 cases per 100,000 people. GBS can occur in children, but the annual incidences of GBS rise with age increasing^18^. GBS may cause life-threatening condition. A cohort study found that the mortality rate of GBS was 3.9%^20^. In addition, GBS is an immune-mediated peripheral neuropathy. Abnormal autoimmune responses triggered by a preceding infection attack the peripheral nerves^18^. Molecular mimicry exists between microbial antigens and ganglioside epitopes. Antiganglioside antibodies are involved in the pathogenesis of GBS^19,21^. There are links between the clinical manifestations of GBS and various antiganglioside antibodies. Anti-GQ1b antibody is associated with classic Miller–Fisher syndrome (MFS), and anti-GM1 antibody is related with AMAN^19^. In ultrastructural studies, macrophage-induced demyelination was observed in patients with AIDP^22^.

**Figure 1:**
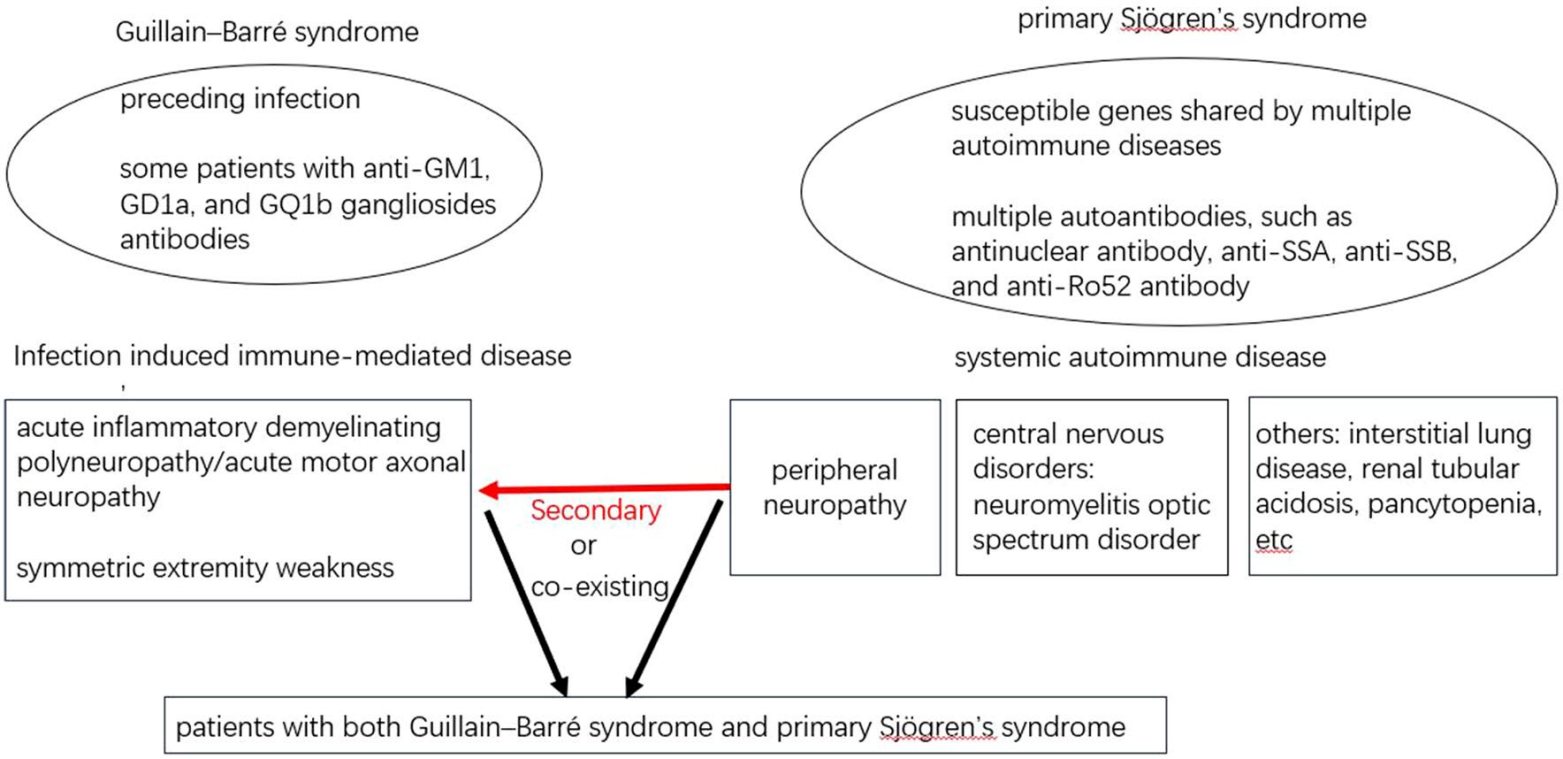
The typical signs and symptoms characterizing Guillain–Barré syndrome and primary Sjögren’s syndrome.

In patients with primary SS, anti-ganglion neuron antibodies are associated with sensory neuropathy^23^. However, it is unclear if GBS, an autoantibody-related disease, presents unique clinical features in patients with primary SS, a systemic autoimmune disease characterized by B cell activation and multiple autoantibody production. When a patient suffered from both GBS and primary SS, it is still uncertain about their relationship, the GBS is secondary to primary SS or they are co-exiting (Fig. 1). In the present study, we performed a retrospective analysis of the clinical features of GBS associated with primary SS at a Chinese tertiary medical center. The study may help clinicians by raising awareness of the simultaneous presentation of GBS and primary SS in clinical practice.

## Methods

**Figure 2:**
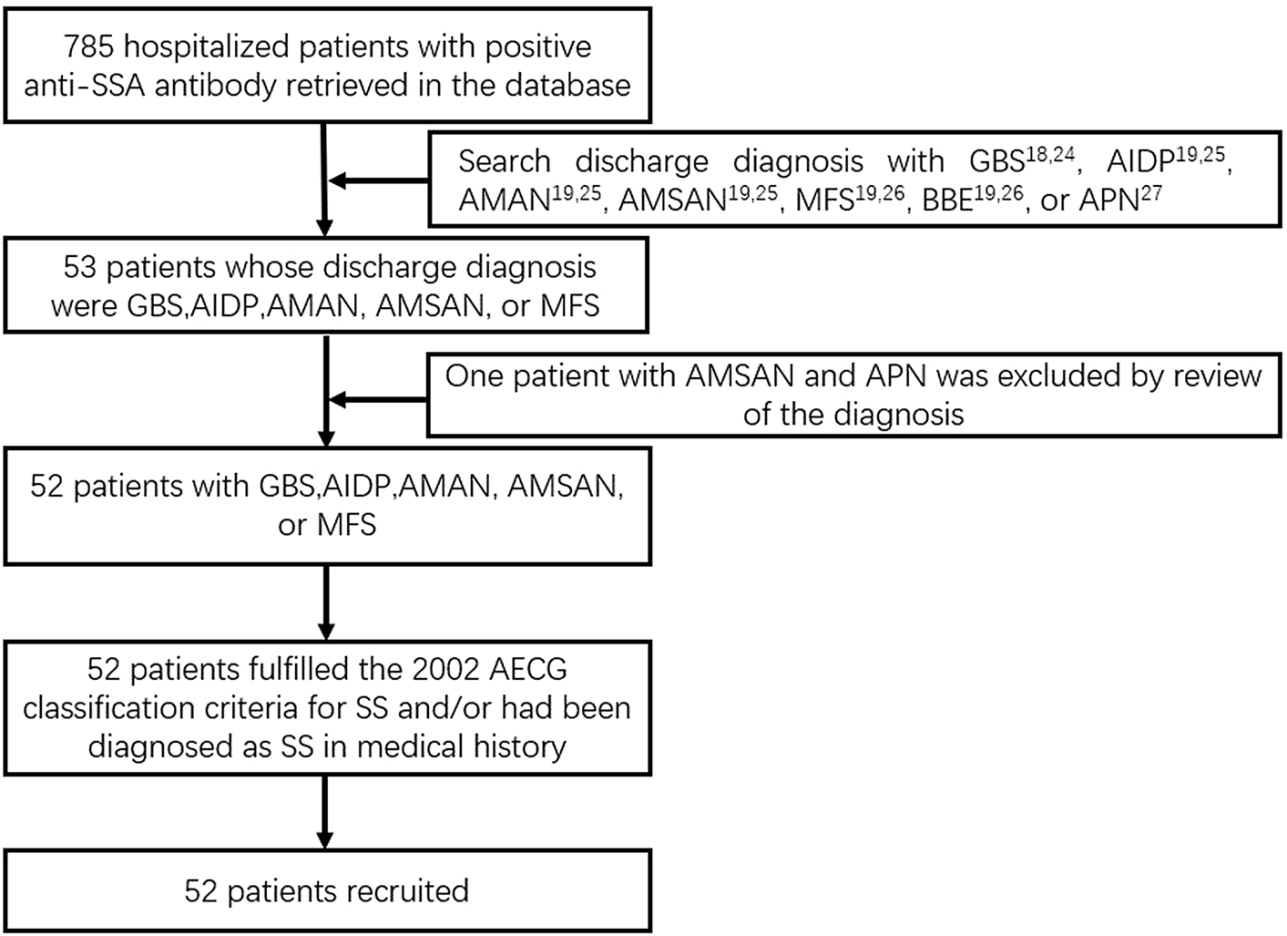
The flowchart of patients included in the research. *GBS* Guillain–Barré syndrome, *AIDP* acute inflammatory demyelinating polyneuropathy, *AMAN* acute motor axonal neuropathy, *AMSAN* acute motor sensory axonal polyneuropathy, *MFS* Miller–Fisher syndrome, *BBE* Bickerstaff brainstem encephalitis, *APN* acute panautonomic neuropath, *AECG* American-European Consensus Group, *SS* Sjögren’s syndrome.

This study was approved by the Ethics Committee of the Beijing Tiantan Hospital. The research had been performed in accordance with the Declaration of Helsinki. The requirement for informed consent was waived by Ethics Committee of the Beijing Tiantan Hospital owing to the retrospective nature of the study. De-identified inpatient clinical data were provided by the Information Center of the Beijing Tiantan Hospital. Data of patients with positive anti-SSA antibodies who were hospitalized in the Beijing Tiantan Hospital between December 2011 and May 2020 were retrieved. A total of 785 patients were included in this study. By reviewing medical records, we enrolled patients with a discharge diagnosis of GBS, AIDP, AMAN, acute motor sensory axonal polyneuropathy (AMSAN), MFS, or acute panautonomic neuropathy (APN). A total of 53 patients were identified. GBS was diagnosed when the patient fulfilled the 1990 revised criteria proposed by the National Institute of Neurological Disorders and Stroke^18,24^. The diagnoses of AIDP, AMAN, and AMSAN were based on the neurophysiological subtype classification criteria suggested by Uncini et al.^19,25^. MFS manifests as ophthalmoparesis, areflexia, and ataxia. Patients with Bickerstaff brainstem encephalitis have encephalopathy and hyperreflexia in addition to the features of MFS^19,26^. The diagnosis of APN is based on clinical and electrophysiological features^27^. The diagnoses of GBS subtypes were confirmed by expert neurologists during hospitalization. Dr. Hua Pan reviewed the diagnoses, and one patient was excluded because of nerve conduction features. Involvement of the respiratory muscle was defined if patients had dyspnea or respiratory failure detected by arterial blood gas analysis, and other possible causes were excluded. All 52 patients fulfilled the 2002 American-European Consensus Group classification criteria for primary SS based on clinical and laboratory data and/or had been diagnosed with primary SS based on medical history^28^ or fulfill the 2016 American College of Rheumatology/European League Against Rheumatism classification criteria for primary SS^29^. Finally, 52 patients were identified and included in this study (Fig. 2).

The methods for nerve conduction studies in our hospital have been described previously^30^. Each patient underwent sensory nerve conduction studies; orthodromic for the median, ulnar, medial plantar, and superficial fibular nerves; antidromic for the sural nerve; and motor nerve conduction studies for the median, ulnar, tibial, and fibular nerves. All studies were performed via surface recordings using conventional techniques. The nerves were stimulated using 0.1 ms electrical pulses with supramaximal intensity defined as a stimulus 20% greater than a stimulus that was just sufficient to elicit maximum amplitude compound muscle action potentials or sensory nerve action potentials.

Autoantibodies were tested by the clinical laboratory of the Beijing Tiantan Hospital using EUROIMMUN, Hangzhou, China. Antinuclear antibody (ANA), anti-parietal cell antibody, and anti-mitochondrial antibody were detected by indirect immunofluorescence; anti-SSA, anti-Ro52, anti-SSB, anti-centromere antibody (ACA), anti-PM-Scl antibody, anti-nRNP, and Anti-mitochondrial antibody M2 subtype (AMA-M2) were examined by immunoblot assay; meanwhile, anti-thyroid peroxidase (TPO) and anti-thyroglobulin (TG) autoantibodies were detected by enzyme-linked immunosorbent assay.

### Statistical analysis

We used SPSS 24.0 to perform all statistical analyses. Categorical variables were expressed as numbers or percentages. The Kolmogorov–Smirnov test was used to examine whether continuous variables were normally distributed. Normally distributed continuous variables are expressed as means. The median was used for data with non-normal distribution.

## Results

For the 52 patients, their median age was 59 (18–74) years old. Among them, 27 patients were males and 25 were females (Table 2). With regard to the clinical manifestations of primary SS, hematological abnormality was observed in 7 patients including 2 patients with anemia, 1 patient with both anemia and leukopenia, 2 patients with leukopenia, 1 patient with both leukopenia and thrombocytopenia, and 1 patient with both thrombocytopenia and anemia. Concomitant primary biliary cholangitis (PBC) was observed in four patients, and interstitial lung disease was observed in two patients. Two patients showed articular involvement. The diagnosis of Hashimoto’s thyroiditis (HT) was definite in 11.5% (6/52) of patients with primary SS.

**Table 2:** The demographic characteristics of 52 patients.

Clinical characteristics	N = 52 n/median
Age	59
Female	25
Hematological involvement	7
Anemia	2
Anemia and leukopenia	1
Leukopenia	2
Leukopenia and thrombocytopenia	1
Thrombocytopenia and anemia	1
Primary biliary cholangitis	4
Interstitial lung disease	2
Articular involvement	2
Hashimoto’s thyroiditis	6
Preceding infection	42
Respiratory infections	28
Gastrointestinal infections	10
Concurrent infections of the respiratory and gastrointestinal tracts	3
Parotitis	1

**Figure 3:**
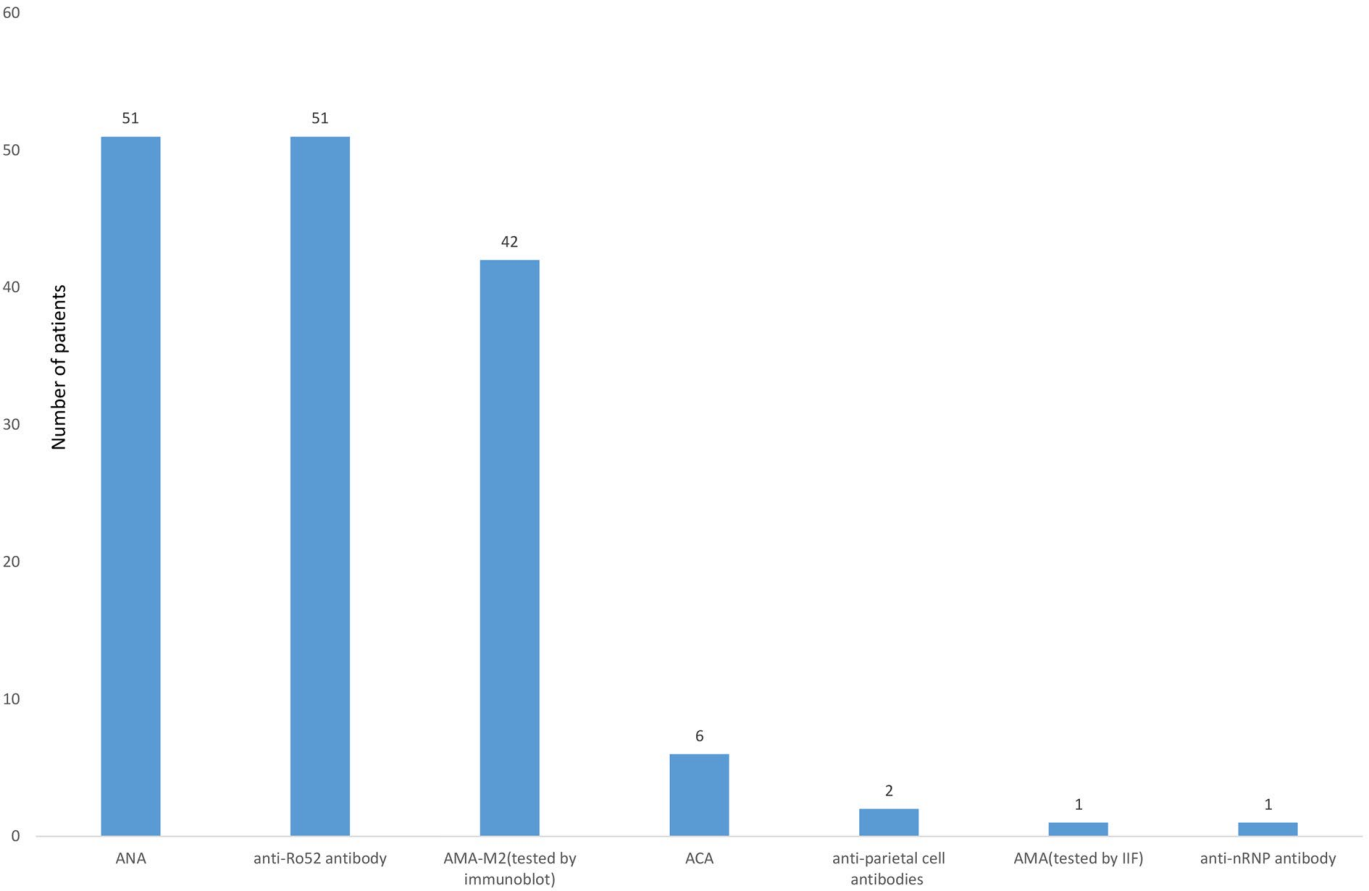
The frequency of positive autoantibodies in 52 patients. *ANA* antinuclear antibody, *AMA-M2* anti-mitochondrial antibody M2 subtype, *ACA* anti-centromere antibody, *AMA* antimitochondrial antibody, *Anti-nRNP antibody* anti-nuclear ribonucleoprotein antibody, *IIF* indirect immunofluorescence.

As shown in Fig. 3, in addition to one patient with negative ANA results, the remaining 51 patients had positive ANA results. Anti-SSB antibody test results were negative in all 52 patients. A positive anti-Ro52 antibody result was observed in 51 patients of 52 patients. Positive AMA-M2 antibodies were detected in 42 patients (80.8%). All patients had negative rheumatoid factors. Positive ACA was observed in six patients. Anti-parietal cell antibodies were observed in two patients. Anti-mitochondrial and anti-nRNP antibody were detected in one patient respectively. Among 49 patients who tested anti-TG autoantibody and anti-TPO autoantibody, 47 (96%) patients had elevated anti-TG autoantibodies, and 48 (98%) patients had anti-TPO autoantibodies.

Preceding infection was recorded in 42 patients, 28 patients had respiratory infections, 10 patients had gastrointestinal infections, and 3 patients had concurrent infections of the respiratory and gastrointestinal tracts. One patient had parotitis before GBS onset.

As shown in Table 3, at the onset of disease, limb weakness occurred in 33 patients, numbness was observed in 28 patients, cranial neuropathies occurred in 12 patients and pain occurred in 10 patients. Some patients initially presented more than one symptom at the onset (Table 3).

**Table 3:** Initial symptoms of 52 patients.

Initial symptoms	Cases (%)
Limb weakness alone	13 (25.0%)
Limb weakness and numbness	8 (15.4%)
Numbness alone	7 (13.5%)
Cranial neuropathies alone	5 (9.6%)
Limb weakness and numbness and cranial neuropathies	4 (7.7%)
Numbness and pain	3 (5.8%)
Limb weakness and numbness and pain	3 (5.8%)
Limb weakness and paresthesia	2 (3.9%)
Pain alone	1 (1.9%)
Ataxia and pain	1 (1.9%)
Limb weakness and numbness and cranial neuropathies and ataxia	1 (1.9%)
Numbness and cranial neuropathies and ataxia	1 (1.9%)
Limb weakness and paresthesia and pain	1 (1.9%)
Limb weakness and cranial neuropathies	1 (1.9%)
Pain and numbness and autonomic dysfunction (have difficulty urinating or defecating)	1 (1.9%)

During the disease course, 51 of 52 patients had hyporeflexia or areflexia. A total of 49 patients presented with limb weakness. Numbness was observed in 39 patients. A total of 35 patients had cranial nerve injuries (Table 4). Facial nerve involvement was the most common manifestation, followed by involvement of the oculomotor and glossopharyngeal nerves. Trigeminal neuropathy was observed in three patients. Concerning the number of cranial nerves involved in a patient, 13 patients had isolated cranial nerve involvement. Two and three types of cranial nerve involvement were seen in seven and six patients, respectively. Five patients had involvement of four types of cranial nerves. In particular, two patients had involvement of five cranial nerves and two patients had involvement of six cranial nerves.

**Table 4:** The involvement of cranial nerves in 35 patients.

The cranial nerve involved	Number of patients (percentage)
Facial nerve	22 (62.9%)
Oculomotor nerve	15 (42.9%)
Glossopharyngeal nerve	14 (40%)
Abducens nerve	13 (37.1%)
Vagus nerve	12 (34.3%)
Trochlear nerve	5 (14.3%)
Trigeminal nerve	3 (8.6%)
Accessory nerve	2 (5.7%)
Auditory nerve	1 (2.9%)

Twenty-one patients experienced pain during the disease course. Twenty patients presented autonomic dysfunctions. Ataxia was observed in 10 patients. Thirteen patients suffered from respiratory muscle involvement. Among them, four patients had respiratory failure, and three patients were treated with mechanical ventilation. We used the GBS disability score^31^ to assess the functional status of patients in the peak phase according to clinical data. As shown in Table 5, a total of four (7.7%) patients were able to walk independently; 28 (53.8%) patients were unable to walk unaided; 15 (28.8%) patients were bedridden or wheelchair-bound; and three (5.8%) patients required mechanical ventilation. In addition, when the patients were admitted, the muscle strength of the upper and lower limbs on both sides was measured and recorded. The minimum muscle strength of patients on admission was recorded. Muscle strength of three patients was graded as 0; muscle strength of seven patients was graded as 1; muscle strength of eight patients was graded as 2; muscle strength of nine patients was graded as 3; muscle strength of one patient was graded as 4-; and muscle strength of 24 patients was graded as 4–5. Forty-three patients received treatment before admission to our hospital.

**Table 5:** The GBS disability score of 52 patients.

GBS disability score	Numbers of patients n (%)
Healthy, 0	0 (0)
Minor symptoms but able to run, 1	2 (3.8)
Able to walk independently, unable to run, 2	4 (7.7)
Not able to walk independently for at least 10 m, 3	28 (53.8)
Bedridden or wheelchair bound, 4	15 (28.8)
Mechanically ventilated for at least part of the day, 5	3 (5.8)
Dead due to Guillain–Barré syndrome,6	0 (0)

In terms of GBS subtypes, 23 patients were classified as having AIDP; 12 patients were categorized as having an AMAN; 10 patients had MFS; and seven patients had AMSAN. In our study, both lumbar puncture and nerve conduction studies were performed in 46 patients, and the rest six patients underwent either cerebrospinal fluid (CSF) analysis or nerve conduction studies. Lumbar puncture was performed in 48 patients (Table 6). Albuminocytologic dissociation was observed in the CSF of 33 patients. A total of 50 patients underwent electromyography. Axonal injury was found in 16 patients, demyelinating changes were identified in 13 patients, and both axonal injury and demyelinating changes were detected in 3 patients. Ten patients were diagnosed as non-specific abnormalities because their electromyography could not be categorized. Eight patients did not detect neurogenic injury by nerve conduction studies.

**Table 6:** The cerebrospinal fluid results of 48 patients underwent lumbar puncture.

	N = 48 n
Increased CSF protein	34
Normal CSF cell count	46
Cytoalbuminological dissociation	33

A total of 46 patients received intravenous immunoglobulin (IVIG) monotherapy. Combination therapy of IVIG and glucocorticoids (GCs) was administered to five patients. One patient was administered neurotrophic treatment without other treatments including IVIG, GCs or plasma exchange. Except one patient, 51 of 52 patients showed improvement of clinical symptoms after treatment during hospitalization.

## Discussion

In the present study, we reported 52 patients with primary SS got GBS, their median age was 59 years and the male-to-female sex ratio was 1.1 to 1. It is interesting that male was slightly more common than female in our patients with both GBS and primary SS. It has been well established that female to male ratio in patients with SS is about 9:1^32^ or even higher^33^. The gender distribution is about 1:1 in patients with GBS^32,34^. If an organ involvement was secondary to primary SS, its gender distribution should be similar to that of primary SS with predominantly higher proportion of female. It was reported that in primary SS patients with PNS involvements including sensory and/or motor neuropathy that were caused by abnormal autoimmune reaction of primary SS, the proportion of male was 8.1% without significant difference from that in patient with primary SS alone^6^.The gender ratio of about 1:1 in our patients is in accordance with that in GBS population and supports the notion that these two autoimmune diseases are co-existing.

In our study, most patients had a recent respiratory or gastrointestinal infection. Limb weakness was the most common symptom at disease onset. Hyporeflexia/areflexia, numbness, cranial nerve injury, and pain were very common in the disease course. AIDP and AMAN were the major GBS phenotypes. Albuminocytologic dissociation detected by CSF analysis was observed in most patients. These findings are in accordance with typical clinical features of GBS^18,19^. During the disease course, cranial nerve impairment occurred in 35 of 52 patients (67.3%), and the frequency of facial, oculomotor, glossopharyngeal and abduces nerves involvements were 62.9%, 42.9%, 40%, and 37.1% respectively (Table 4). Furthermore, the involvement of multiple cranial nerves was found in 22 (62.9%) of 35 patients. This finding was similar to literature which reported that the involvement of cranial nerve is observed in 62.3% of GBS patients, and 65.8% of those with cranial neuropathy showed multiple cranial nerves were involved^35^. Bulbar palsy and facial nerve palsy were presented in 49.2% and 46% of GBS patients, respectively^35^. However, it has been reported that the prevalence of cranial neuropathies in primary SS with neurologic manifestations was 19.5% (16/82)^36^ and the frequency of facial nerves involvement was 4.9% (4/82)^36^. Another paper reported the frequency of cranial or phrenic nerve impairment in primary SS patients with neurological manifestations was 19.7% (47/238)^7^. It was also reported that trigeminal neuropathy was the most common cranial nerve involvement in patients with primary SS^3^. As our patients showed much higher frequency of cranial nerve involvement than that reported in primary SS alone and infrequency of trigeminal neuropathy, we speculated that GBS patients with primary SS may develop cranial nerve neuropathies by basic pathological mechanisms of GBS. Along with the finding that all of our patients presented multiple autoantibodies, GBS is likely induced by an infection in patients with primary SS, and multiple autoimmune diseases tend to co-exist in an individual.

In 11 patients with both primary SS and GBS reported in the literature^9–17^ (Table 1), five patients were classified to have axonal injury and four patients showed demyelinating changes. In our study, the frequency of axonal damage was also slightly higher than that of demyelinating changes on electromyography. All of 11 patients reported in literature recovered or improved, however, 9 of them applied GCs or intensive immunosuppressive agents. In our study, 51 of 52 patients improved during hospitalization and only 5 of them applied GCs. Thus, most patients with GBS and primary SS showed benign disease course and the diagnosis of primary SS dose not necessitate prompt immunosuppressive agents including GCs which may be preserved as second-line treatment for refractory cases.

There were several limitations. Firstly, most of our patients were hospitalized in department of neurology. The primary SS was diagnosed based on the medical history, symptom, past tests, and autoantibodies. The standardized screening for primary SS was not performed. Patient with positive anti-SSB antibody but without anti-SSA antibody were not included in the study, thus we might miss some patients. Secondly, many autoantibodies in this research were tested by immunoblot assay which have lower specificity, thus false positive results might present in some patients. Finally, although plasmapheresis is available in China, none of patients was treated with plasmapheresis in this study due to poor economic status of patients or shortage of blood plasma supply. In the future, prospective studies with larger sample sizes are required to validate the clinical features of GBS in patients with primary SS.

### Supplementary Information

Supplementary Table 1.

## Data Availability

All data generated or analysed during this study are included in this published article and a supplemental table.
